# Characterizing the Local Material Properties of Different Fe–C–Cr-Steels by Using Deep Rolled Single Tracks

**DOI:** 10.3390/ma13214987

**Published:** 2020-11-05

**Authors:** Nicole Wielki, Noémie Heinz, Daniel Meyer

**Affiliations:** 1 Faculty of Production Engineering, University of Bremen and MAPEX Center for Materials and Processes, Badgasteiner Str. 1, 28359 Bremen, Germany; noeheinz@uni-bremen.de (N.H.); dmeyer@iwt-bremen.de (D.M.); 2 IWT Leibniz-Institute for Materials Engineering, Badgasteiner Str. 3, 28359 Bremen, Germany

**Keywords:** plastic deformation, deep rolling, material characterization

## Abstract

As part of a novel method for material development, deep rolling was used in this work to characterize the mechanical properties of macroscopic specimens of C45 (AISI 1045), S235 (AISI 1015), and 100Cr6 (AISI 52100) in various heat treatment states. Deep rolling is conventionally used to enhance surface and subsurface properties by reducing the surface roughness, introducing compressive residual stresses, and strain hardening. In the context of this work, it was utilized to determine material-specific variables via a mechanically applied load. For that purpose, the geometries of individual deep rolled tracks were measured. In dependence of the process parameters such as deep rolling pressure and tool size, the track geometry, i.e., the specific track depth, was for the first time compared for different materials. A functional relationship identified between the specific track depth and the material state dependent hardness forms the basis for a future characterization of the properties of alloy compositions belonging to the Fe–C–Cr system. Since deep rolling is performed in the same clamping as machining operations, hardness alterations could easily be determined at different points in the process chain using an optical in-process measurement of track geometries in the future.

## 1. Introduction

Increasing demands of various industries such as the aerospace industry [1] on the performance of, e.g., lightweight parts, require the development of specially adapted, application oriented materials. In order to accelerate the material development process, which is conventionally time and cost intensive, Mädler and Ellendt proposed an approach based on the determination of fast and easily measurable material-specific variables [2,3]. By describing the reactions of the material to, e.g., mechanical or thermal loads, these variables allow conclusions about classic material properties, as indicated for micro samples by the investigations of Steinbacher et al. [4].

Deep rolling (also known as burnishing) is a process conventionally applied as a processing or post-processing operation to enhance surface and subsurface properties by reducing the surface roughness and introducing compressive residual stresses [5,6]. It is used, e.g., in the aviation (cf. [7]) and automotive industries [8] and aims to improve component fatigue strength and lifetime due to strain hardening and compressive residual stresses [9,10,11,12]. Crack formation is delayed by the reduced surface roughness and strain hardening and crack propagation are slowed down by compressive residual stresses [13].

Deep rolling investigations by Magalhaes et al. on various heat-treated samples of the material AISI 1060 have shown that the influence of the process parameters on, e.g., the surface roughness, differs depending on the material hardness [14]. The softer the material, the more likely it is that the increase in track depths with increasing deep rolling pressure or number of rollovers will lead to an increase in surface roughness. Regarding a constant deep rolling pressure of 200 bar, Magalhaes et al. determined a reduction of surface roughness for all material states after one rollover. While no remarkable change in surface roughness was determined for the subcritical annealed or the quenched and tempered state after three rollovers, the fully annealed material state exhibited a higher surface roughness after three rollovers. [14]

When investigating the surface topography, a different material reaction can be recognized when applying the same combination of process parameters to different material states. Based on this observation, Wielki et al. proposed the use of deep rolling to determine material-specific variables due to a mechanically applied load [15]. They utilized the process to plastically deform 100Cr6 (AISI 52100) samples of different size and heat treatment. Single deep rolled tracks were created on the samples with macroscopic dimensions using different process parameters (varied tool size, varied deep rolling pressure, and single rollover). The track geometry was subsequently characterized by means of track width and track depth. The plastic deformation (tracks) was compared to deformed spherical microscopic samples through the aid of the calculated maximal equivalent stress [15]. Thereby, a parameter-independent description of the plastic deformation became possible for both microscopic and macroscopic samples. Investigations by Kämmler et al. further showed the influences of varying numbers of rollovers (number of contact n) on the residual stresses for quenched and tempered 42CrMo4 (AISI 4140) [16]. Comparing the maximal equivalent stress and the maximal residual stress, a tool diameter-independent process signature was identified. While for the determination of residual stresses Kämmler et al. additionally generated deep rolled areas with overlapping tracks (due to measuring spot size), Meyer et al. showed that using a 0.2 mm microfocus spot enables the determination of residual stresses even under single deep rolled tracks [17]. This microfocus measuring aperture was also used in the investigations of Hettig and Meyer, who focused on the influence of multistage deep rolling on the track geometry and the residual stresses of 42CrMo4 [18]. By varying not only the number of rollovers but also considering the influence of successively using a larger (tool diameter: d_b_ = 13 mm) or a smaller tool (d_b_ = 6 mm), they were able to systematically influence the resulting residual stresses. Using the lager tool first resulted in higher compressive stresses which occurred up to higher distances from the surface.

Thus, the influences of the process parameters on the resulting track geometry were confirmed for defined states of the materials 100Cr6 and 42CrMo4. Depending on the material state (i.e., hardness and toughness), different track geometries were determined when using the same process parameters. This suggests that it should be possible to draw conclusions about certain material properties based on the track geometry in a wide range of materials (steels). Such an approach could be of interest for characterizing the effects of individual process steps (e.g., deep rolling, milling, and grinding) using additionally deep rolled single tracks. Optical measurements would allow for obtaining the relevant information from the single tracks very quickly, so that in-process statements could be made without unclamping the sample.

Since there is still no cross-material comparison of the plastic deformation behavior characterized by deep rolling, it is not clear whether the selected track characterizing parameters are suitable for distinguishing not only different heat treatment conditions, but different alloy compositions. This is addressed in the current work investigating the materials C45 (AISI 1045), S235 (AISI 1015), and 100Cr6 (AISI 52100; different heat treatments and varying compositions of alloying elements).

## 2. Materials and Methods

### 2.1. Materials

**Figure 1:**
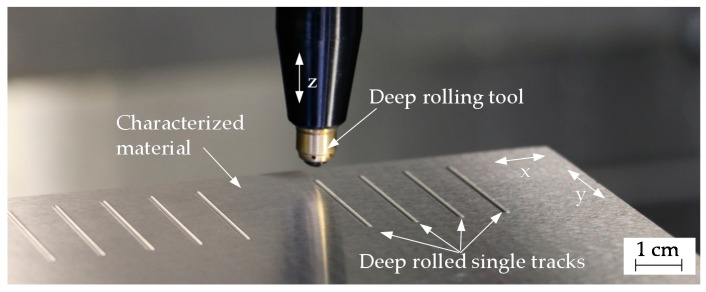
Exemplary presentation of deep rolled single tracks on 100Cr6 SA using a tool with a diameter of 6 mm (varied deep rolling pressure and number of rollovers).

The deep rolling experiments were carried out on macroscopic plane specimens (various dimensions; between 65 mm and 180 mm long and wide; at least 7 mm thick) of three steels (C45 (AISI 1045), S235 (AISI 1015), and 100Cr6 (AISI 52100)), which allowed individual tracks with a length of 20 mm to be deep rolled. Figure 1 exemplarily shows the deep rolling tool with a diameter of 6 mm and different deep rolled single tracks.

Table 1 lists their chemical compositions. While S235 and 100Cr6 were commercially available alloy compositions, C45 MPIE and 100Cr6 MPIE were cast at the “Max-Plank-Institut für Eisenforschung” (MPIE) in Düsseldorf, Germany. In addition to the name-giving alloying elements, these “pure” alloy compositions contained lesser amounts of other elements. It is expected that the differences in alloy composition lead to differences in various material properties, such as hardness, when applying the same heat treatment. Based on the good hardenability assigned to the alloying elements silicon and manganese, lower hardness is expected to result for the MPIE materials. These highly defined materials allow for validating the applicability of the new approach to a wide range of materials with different alloying compositions.

In order to consider both the influences of the additional alloying elements and the influences of different heat treatments, the material 100Cr6 was investigated in a soft annealed (SA) and a hardened and tempered condition (QT1). The latter heat treatment was also performed on the 100Cr6 MPIE samples. The heat treatment (quenched and tempered: QT) of the C45 MPIE was selected to achieve a material hardness comparable to 100Cr6 SA. With the adjusted ferritic pearlitic structure, S235 represents the softest state. All heat treatments are listed in Table 2.

To achieve comparable surface roughness of Ra ≤1 µm, all samples were initially ground (pendular grinding; cutting speed: 30 m/s; feed rate: 10 m/min; depth of cut: 10 µm) before performing deep rolling experiments.

After grinding, the Vickers hardness HV 1 was determined on the surfaces of the samples for each material (state); cf. Table 3 (cf. [19] for procedure). Despite different alloy compositions, similar hardness was achieved for C45 MPIE QT and 100Cr6 SA. The same heat treatment of 100Cr6 MPIE QT1 resulted, as expected, in less than half the hardness of the 100Cr6 QT1.

### 2.2. Methods and Devices

Deep rolling experiments were performed on a 3-axis CNC machining center (DMC 65V by Deckel Maho, DECKEL MAHO-Straße 1, 87459 Pfronten, Germany) utilizing a hydrostatic tool with a spherical ceramic tip Ecoroll HGx (Ecoroll AG Werkzeugtechnik, Celle, Germany). The applied process parameters are summarized in Table 4. Three experiments were performed for each parameter combination.

Since materials of different hardness, which offer different resistances to deformation, were investigated within this work, a large range of forces was applied by using three different tool diameters *d*_b_ = 3, 6 and 13 mm, and varying deep rolling pressures. Comparing the deformation is possible across all tool sizes based on matching maximum equivalent stresses, which are listed in Table 4. The maximum equivalent stress is calculated with the aid of the theoretical deep rolling force *F*_r, theor._ (similar orders of magnitude are marked in bold) for 100Cr6 SA according to von Mises (sphere-plane-contact) [20]. The theoretical deep rolling force was calculated using the functional relationship presented in Equation (1) (cf. [21]).

(1) $$F_{r,\;\mathrm{theor}.}\;=\frac{1}{2}\;\cdot \;p_{r}\;\cdot \;\pi \;\cdot \;d_{b}$$

For calculation, a Poisson’s ratio ν of 0.3 and a Young’s modulus E of 210 GPa were used for the workpiece, and 0.22 and 305 GPa respectively for the tool. In the figures shown below, the results are plotted in relation to the deep rolling pressure.

The deep rolling force *F*_r_ was measured utilizing a piezoelectric 3-component dynamometer (Typ 9257 B, Kistler Instrumente GmbH, Sindelfingen, Germany) equipped by a Kistler charge amplifier Typ 5019 A. A 500 Hz low pass filter was used to reduce the noise of the signal and the sampling rate was selected as 2500 Hz. Averaged over all experiments, the measured deep rolling forces appeared to be about 20% lower than the theoretical forces due to pressure losses in the system.

**Figure 2:**
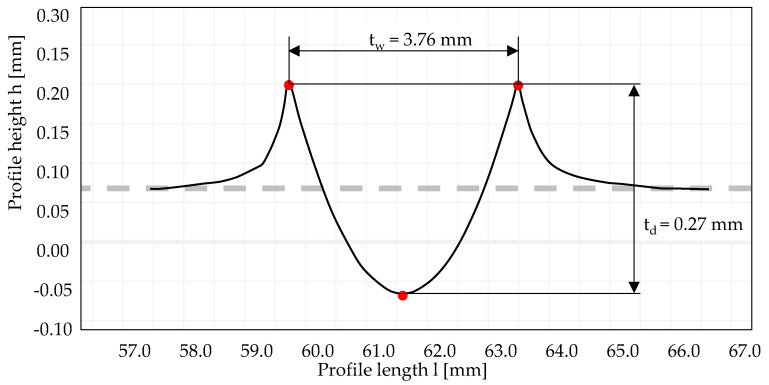
Characterization of the track geometry.

The track geometry was determined utilizing the stationary tactile surface measuring device Surftest SV3200 (Mitutoyo Deutschland GmbH, Neuss, Germany). It enables the measurement of heights up to 500 µm (force 4 mN). The probe tip had a radius of 2 µm and a tip angle of 60°. Determined track geometries were post processed in the software FORMTRACEPAK version 5.523 of Mitutoyo. Figure 2 exemplarily shows a track profile resulting after deep rolling 100Cr6 SA with a pressure of 400 bar and a tool with a diameter of 13 mm (number of rollovers n = 30). As indicated, the variables track width t_w_ and track t_d_ depth were determined within the software.

## 3. Results

The examination of the track depth t_d_ and the track width t_w_ in regard to the input parameters shows an expectable deepening and widening of the track with increasing deep rolling pressure p_r_ and number of rollovers n. For high numbers of rollovers, the track characterizing values tend to converge towards a deep rolling pressure-dependent threshold value. This is in good agreement with the observations of Hettig and Meyer, who attributed the reduced increase in plastic deformation with an increasing number of contacts to the changing contact area [18]. If no more changes in the track geometry are determined, it can be assumed that there is a balance between the material properties and the deep rolling force which influences the surface [18].

**Figure 3:**
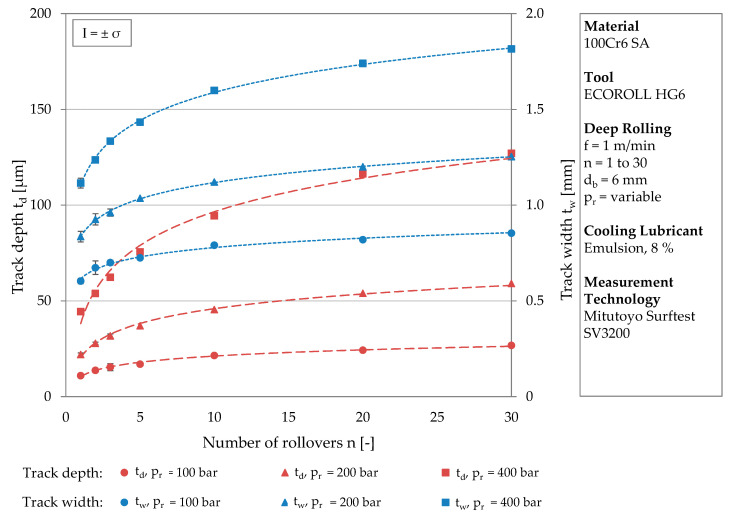
Development of the track depth and the track width at different deep rolling pressures with an increasing number of rollovers.

Figure 3 exemplarily shows the development of track depth and width for the material 100Cr6 SA at a tool diameter of *d*_b_ = 6 mm. On the two y-axes, the track depth and the track width are plotted for various numbers of rollovers (x-axis). The circular icons correspond to the lowest and the square icons to the highest deep rolling pressure. It can be clearly seen that the initial gradient of the trend curve depends on the deep rolling pressure. The geometry of the tracks, therefore, strongly depends on the pressure. The standard deviations are below σ = ± 6.5% for all measurements of track depth and below σ = ± 4.3% for track width. The measured values were each subjected to a logarithmic regression, visualized by the red (track depth) and blue (track width) trend lines. All trend curves have in common that they start with a relatively steep gradient then flatten out as they continue. The curves of the track width have a greater overall gradient, which is due to the spherical geometry of the deep rolling tool.

Table 5 lists the functional relationships of the trend lines plotted Figure 3 and the related coefficients of determination. The trend lines represent the development of the track geometry up to a number of rollovers of *n* = 30 with very good coefficients of determination of at least R² = 0.9446.

Since the trend curves depend on the deep rolling pressure and differ in their positions, they are represented by different functional equations. With increasing deep rolling pressure, both the constant and the multiplication factor of the function increase. Considering the value of the function for one rollover, the higher constant with increasing pressure is reasonable. In this case (*n* = 1), the logarithmic term is set to the value 1 and the constant reflects the higher deformation associated with a higher load. The increasing multiplication factor with higher deep rolling pressure leads to steeper trend lines but comparable proportional increases in geometric sizes. Regarding, e.g., the track depth after one, two, and three rollovers, independently of the deep rolling pressure, increases of approximately 25% (*n* = 1 to *n* = 2) and approximately 14% (*n* = 2 to *n* = 3) were determined.

Although logarithmic functions mathematically do not reach a threshold value, the trend lines do. The influence of the number of rollovers decreases at higher pressures, since the resistance to plastic deformation increases with higher initial deformation due to strain hardening and changing contact conditions.

**Figure 4:**
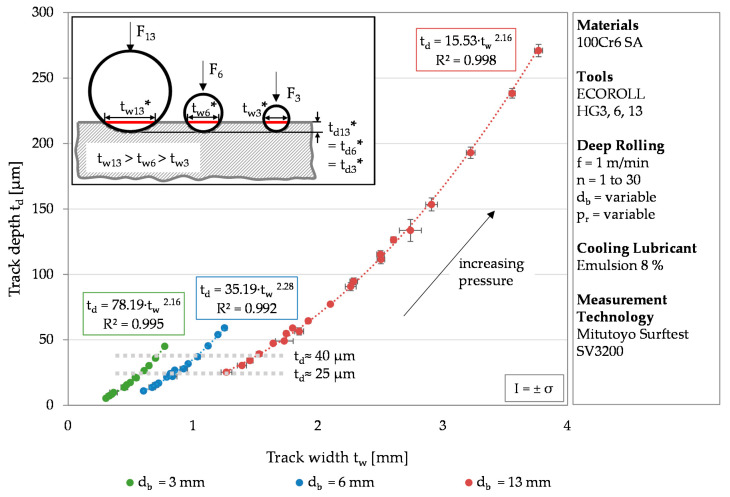
Correlation between the track depth and the track width as a function of the deep rolling pressure and the number of rollovers at different tool diameters.

While Figure 3 the results for one tool diameter, Figure 4 visualizes the track depth over the track width for varying tool diameters. The different tool diameters are color coded. Green represents the results determined after deep rolling using a tool diameter of *d*_b_ = 3 mm, blue using a tool diameter d_b_ = 6 mm, and red using a tool diameter of *d*_b_ = 13 mm. The values were subjected to potency regressions, which all had a coefficient of determination of at least R² = 0.992.

In most cases the standard deviation is below σ = 8.5%. Only the track depths generated with the tool diameter of *d*_b_ = 3 mm for one and two rollovers show higher standard deviations of up to σ = 14.4%. This is due to measurement inaccuracies caused by the small track size. The smaller the tool diameter, the steeper the trend curve (represented by a power function of high determination coefficient R^2^). Regarding a given track depth of 25 or 40 µm (grey dotted line). higher track widths were determined for larger tools (25 µm: t_w,3 mm_ ≈ 0.6 mm, t_w,6 mm_ ≈ 0.9 mm, t_w,13 mm_ ≈ 1.2 mm; 40 µm: t_w,3 mm_ ≈ 0.7 mm, t_w,6 mm_ ≈ 1.1 mm, t_w,13 mm_ ≈ 1.5 mm). In order to achieve similar track depths, a larger tool requires a larger volume of material to be deformed, which in turn requires higher forces. The track depth of 26 µm is reached at a deep rolling force of 232 N for the smallest tool (470 N for the medium tool and 1009 N for the largest tool). This is schematically shown in Figure 4 (F_3_ < F_6_ < F_13_). Since the pictogram visualizes the moment of contact, t_d_* and t_w_* are elasto-plastic values of the track depth and width. For a constant force, a smaller tool diameter leads to higher track depths, since the force acts on a smaller surface. This can be seen for a theoretical deep rolling force of 283 N (d_p_ = 3 and 6 mm) and approximately 1200 N (*d*_p_ = 6 and 13 mm). Resulting track depths were 21 and 11 µm, and 44 and 25 µm, respectively. The different track depths further led to track widths of a similar order of magnitude, which confirms the results obtained by Kämmler et al. for 42CrMo4 [16]. For a deep rolling force of 283 N, track widths of 0.55 µm (*d*_p_ = 3 mm) and 0.60 µm (*d*_p_ = 6 mm) were determined. A deep rolling force of approximately 1200 N results in widths of 1.12 µm (d_p_ = 6 mm) and 1.27 µm (*d*_p_ = 13 mm).

A similar maximum equivalent stress (cf. Table 4) leads to increasing track depths and track widths with increasing tool diameter size. The results illustrate that besides the deep rolling pressure and the number of rollovers, a larger tool diameter also has an increasing impact on the track geometry.

The identified dependency between the track depth and the track width can be explained by the spherical geometry of the deep rolling tool. The correlation determined with the help of the tool-dependent regressions allows one to use only one parameter of the track geometry for further considerations. Investigations of laser deep alloyed specimens by Czotscher et al. revealed a deviating behavior of the track width with increasing hardness, probably caused by the elastic behavior of the tool (an elastic deformation of the tool ball during the deep rolling process) [22]. Since the lateral measurement is more unambiguous compared to the axial evaluation (cf. Figure 1), the track depth will be used for further considerations.

**Figure 5:**
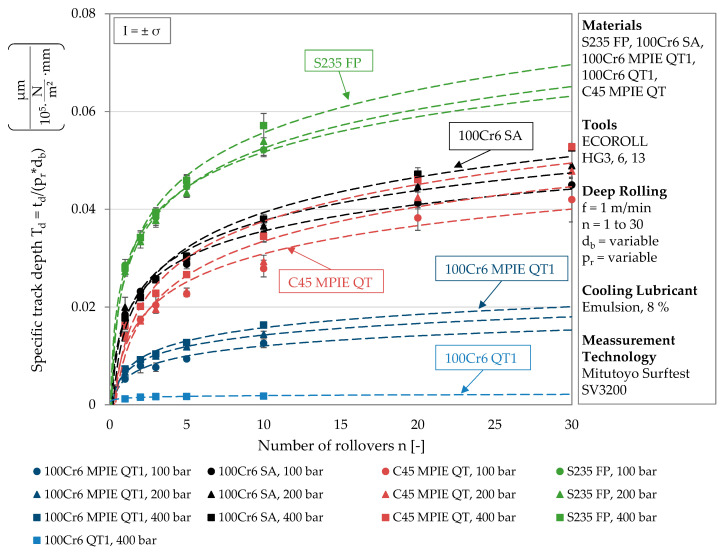
Material-dependent specific track depth as a function of the number of rollovers.

In order to evaluate the track depth in relation to the applied load and the tool size, the specific track depth T_d_ is introduced. It is determined by dividing the track depth by the deep rolling pressure and the tool diameter. Physically, the specific track depth corresponds to a change in area per applied deep rolling force. In this case, the area is the cross-section of the track, which is also represented by the measurement of the track geometry. For the materials investigated within this work, the specific track depth is plotted against the remaining deep rolling parameter n (number of rollovers) in Figure 5. Different material states are separated by different colors to illustrate differences in plastic deformation behavior. The different icons again represent a certain deep rolling pressure, and the values of each deep rolling pressure were subjected to a logarithmic regression. Although the specific track depths do not yield exactly one characteristic curve for each material, the trend curves of one material converge and form a range within which all results are located. As visualized by the different colors, this range varies according to the composition and heat treatment of the respective material.

The trend curves of the specific track depths have high coefficients of determination and are thus well suited to represent a material-specific range of plastic deformation behavior. Both the logarithmic regressions and their coefficients of determination are listed in Table 6. Regarding the constants of the logarithmic regressions, almost identical values can be observed for each material state.

The lower the hardness of the material, the higher are the constant and the position of the specific range. As discussed for Figure 3, here too an increase in the deep rolling pressure correlates with an increasing multiplication factor, which causes the trend curve to steepen and approach a higher threshold value. This can be traced back to the pressure independent proportional deepening of the track with increasing number of rollovers, which results in higher absolute values at higher deep rolling pressure. This effect remains obtainable after the division performed to determine the specific track depth. Regarding the determined multiplication factors, material condition-dependent differences can be identified. While the multiplication factor decreases by 10% from 400 bar to 200 and 100 bar for S235 FP, a higher decrease of around 15% can be observed for 100Cr6 SA and 100Cr6 MPIE QT1. C45 MPIE QT differs from the observation of an approximately constant factor change. The multiplication factor determined at 200 bar is 6% and the factor determined at 100 bar is 23% smaller than the next larger value. It can be concluded that changes in the deformation behavior due to higher loads depend on the material condition and that the specific track depth is suitable for determining this.

Within Figure 5 the position of the characteristic range correlates with the hardness of the materials. Thus, the highest values of plastic deformation were determined for S235 FP, which exhibited the lowest hardness according to Table 3. Although 100Cr6 SA and 100Cr6 QT1 have the same alloy composition, there are large differences in the specific track depth. At a pressure of 400 bar in combination with ten rollovers, the specific track depth of 100Cr6 SA is twenty-one times higher compared to 100Cr6 QT1. While annealing resulted in a low hardness of 204 ± 9 HV for 100Cr6 SA, the material state 100Cr6 QT1 exhibited a four-times higher hardness of 833 ± 8 HV due to low temperature tempering, which is associated with little lattice distortion and remaining austenite. Measurable track geometries were only achieved for the highest deep rolling pressure of *p*_w_ = 400 bar.

Regarding the coefficient of determination listed in Table 6, the high hardness and the corresponding low plastic deformation of this material state resulted in higher deviations of values compared to the other material states. At the same deep rolling pressure, compared to 100Cr6 QT1, a six-times higher specific track depth was determined for material 100Cr6 MPIE QT1. The lower proportions of silicon and manganese in the alloy composition led to a more than two-times lower hardness, which in turn resulted in larger track geometries. The hardness and the characteristic ranges of plastic deformation of C45 MPIE QT and 100Cr6 SA are comparable, although C45 MPIE QT contains less than half as much carbon and significantly less silicon and manganese, which are assigned a good hardenability. However, quenching and tempering of C45 QT and soft annealing of 100Cr6 SA resulted in similar hardness and plastic deformation behavior.

## 4. Discussion

The specific track depth T_d_ allows a differentiation of the material states investigated in this work. By determining the specific track depth based on the published data from Kämmler et al. [16] and Wielki et al. [15], its suitability for material characterization was further investigated. Table 7 lists the additionally considered material states (material composition and heat treatment) and the hardness values resulting after heat treatment. Since only HRC hardness was available, corresponding HV values were calculated based on a conversion table [23].

**Figure 6:**
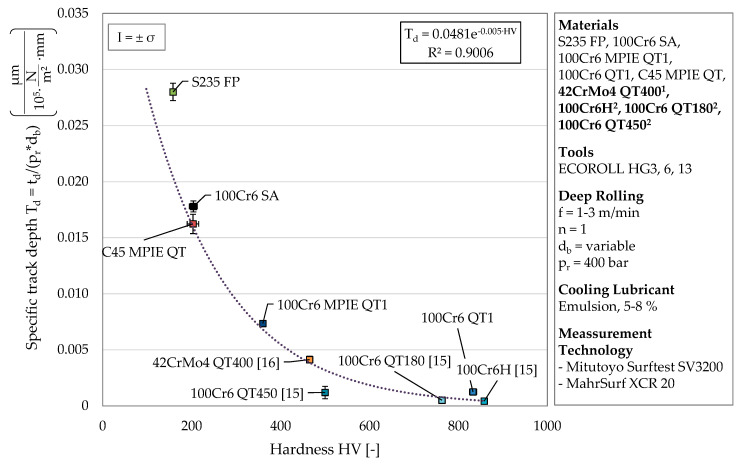
Development of the specific track depth T_d_ with increasing hardness change (^1^values according to [16], ^2^ depths corresponding to the track widths presented in [15]).

Since the material-dependent range is defined by lines of different deep rolling pressure, values were compared for a constant deep rolling pressure of 400 bar and a number of rollovers of *n* = 1. Figure 6 the determined specific track depths in dependence of the corresponding material hardness. Similarly to Figure 5 and as expected, a decreasing plastic deformation is observable for increasing hardness values. The material states additionally presented appear at high hardness values between 100Cr6 MPIE QT1 and 100Cr6 QT1.

The small deformations occurring at high hardness values (above 465 HV) are prone to measurement uncertainties and thus have to be discussed with care. Nevertheless, the determined specific track depths can be related to the material hardness with a coefficient of determination of R^2^ = 0.9006. The exponential relationship presented shows the convergence to a deformation of zero with increasing hardness. Based on the results, it is not yet possible to make a statement about the behavior of the related track depth for hardness significantly lower than considered. Nevertheless, the correlation found could form the basis for a future determination of the material hardness of new material states and alloy compositions belonging to the Fe–C–Cr system. In contrast to the current procedure for determining material hardness, the presented approach could enable a hardness measurement directly on the machine tool and at different points in the process chain when using an optical in-process measurement of track geometries.

Thereby, e.g., strain hardening effects could be quantified by deep rolled single tracks at non-functional surfaces. The possibility of deep rolling to vary the strain rate within a certain range further allows statements about the deformation behavior beyond the quasi-static hardness measurement. Using a mechanically mounted deep rolling tool, the information about the penetration depth could directly be revealed regarding the displacement of the tool.

## 5. Conclusions

This paper focuses on the investigation of deep rolled single tracks in order to characterize the plastic deformation behavior of different Fe–C–Cr-steels. By applying a similar process parameter to various material states (composition and heat treatment), different material behavior can be revealed regarding the resulting track depth and track width. Within this work, a cross-material comparison was carried out for the first time by analyzing the materials C45, 100Cr6, and S235. Besides the parameters describing the track geometry, the specific track depth T_d_ was introduced and considered for the first time. The procedure carried out on these materials was validated by the additional consideration of the track geometries obtained in previous studies (42CrMo4, 100Cr6 further heat treatments). Major new findings of this work are:

- The consideration of up to 30 rollovers supports the theory of a material-dependent maximum achievable plastic deformation, which was assumed regarding previous investigations with *n* = 1−3.
- Determining the specific track depth T_d_ allows a distinction between different material states and revealed the influence of the alloy composition. In addition to a hardness-dependent positioning of the resulting variables within the developed diagram, the slopes of trend lines calculated with a high coefficient of determination indicate the strain hardening behaviors of the investigated materials.
- For constant process parameters, an exponential relationship between specific track depth and the material hardness could be shown, taking into account not only the five material states investigated within this work but also results determined in previous studies by Kämmler et al. [16] and Wielki et al. [15]. This could enable the calculation of the material hardness directly on the machine tool at different points of a process chain based on individual deep-rolled and optically analyzed tracks for material states of the Fe–C–Cr alloy system to be investigated in future.
- Placing another single deep rolling track in a deep rolled area or on a surface which has been processed using other machining operations (turning, grinding) and correlating the geometry of the track with the properties of the (e.g., strain hardened) material could reveal the effects of the process steps
- Due to the adjustable deep rolling velocity, additional statements on the strain rate-dependent material behavior could be made.

## Figures and Tables

**Table 1:** Chemical composition in wt.% of the material investigated within this work according to manufacturer’s specifications and in-house analysis (100Cr6: DNIPROSPEZSTAL PRAG. Prague Czech Republic, 2017; C45 MPIE and 100Cr6 MPIE: Max-Plank-Institut für Eisenforschung, Düsseldorf, Germany; S235: elemental analysis at Leibniz-Institute for Materials Engineering, Bremen, Germany).

	**C**	**Si**		**Mn**	**P**	**S**	**Cr**		**Ni**	**Mo**	**Al**
**C45 MPIE**	0.457	<0.002		0.0384	0.0021	0.0032	0.0124		-	-	-
**S235**	0.078	0.19		0.57	0.014	0.013	0.19		0.20	0.062	0.018
**100Cr6**	0.98	0.26		0.31	0.016	0.009	1.43		0.14	0.02	0.025
**100Cr6 MPIE**	1.03	0.0026		0.0394	0.0028	0.0031	1.60		-	-	-
	**Cu**	**Ca**	**Ti**	**H**	**O**	**Fe**	**N**	**Co**	**V**	**As**	**Sn**
**C45 MPIE**	-	-	-	-	0.0031	Bal.	-	-	-	-	-
**S235**	0.21	-	0.024	-	-	-	0.016	0.010	< 0.01	0.007	0.011
**100Cr6**	0.14	0.0006	0.002	0.0001	-	-	-	-	-	-	-
**100Cr6 MPIE**	-	-	-	-	-	Bal.	-	-	-	-	-

**Table 2:** Heat treatments.

		Hardening Parameter	Quenching Parameter	Tempering Parameter
**C45 MPIE**	QT	650 °C (4 h) in vacuum followed by 850 °C (1 h) in a salt bath	1. Vacuum 2. Water	550 °C (2 h) in vacuum
**S235**	FP	900 °C (1 h) in a salt bath	Atmosphere	-
**100Cr6**	SA	600 °C (30 min) in vacuum followed by 800 °C (5 h) in vacuum and 690 °C (5 h) in vacuum	Furnace cooling	-
	QT1	850 °C (2−3 h) in vacuum	Oil 60 °C	180 °C (2 h) in atmosphere
**100Cr6 MPIE**	QT1	850 °C (2−3 h) in vacuum	Oil 60 °C	180 °C (2 h) in atmosphere

**Table 3:** Material hardness resulting from the heat treatments listed in Table 2.

		Hardness HV 1
**C45 MPIE**	QT	203 ± 13
**S235**	FP	158 ± 3
**100Cr6**	SA	204 ± 9
	QT1	833 ± 8
**100Cr6 MPIE**	QT1	360 ± 5

**Table 4:** Process parameters of the deep rolling process to generate single deep rolled tracks.

Component	Values								
Tool diameter *d*_b_ [mm]	3			6			13		
Deep rolling pressure *p*_r_ [bar]	100	200	400	100	200	400	100	200	400
Theoretical deep rolling force *F*_r, theor._ [N]	71	141	283	283	565	1131	1327	2655	5309
Max. equivalent stress σ_vgl_ [MPa]	2924	3684	4641	2924	3684	4641	2924	3684	4641
Number of rollovers n [-]	1, 2, 3, 5, 7, 10, (20 and 30 only for d_b_ = 6 mm, 13 mm at C45 MPIE QT and 100Cr6 SA)								
Rolling speed v_r_ [mm/min]	100								
Lubricant	rhenus TS 440 as 8%-emulsion								

**Table 5:** Logarithmic regressions for n ≥ 1 of the track depths and the track widths in Figure 3.

Deep Rolling Pressure	Logarithmic Regressions	Coefficient of Determination R²
400 bar	t_d_ = 25.365 ln(n) + 38.158	0.9860
200 bar	t_d_ = 11.083 ln(n) + 20.399	0.9446
100 bar	t_d_ = 4.6589 ln(n) + 10.457	0.9920
400 bar	t_w_ = 0.2108 ln(n) + 1.1034	0.9987
200 bar	t_w_ = 0.1223 ln(n) + 0.8362	0.9990
100 bar	t_w_ = 0.0707 ln(n) + 0.6157	0.9894

**Table 6:** Logarithmic regressions for *n* ≥ 1 of the specific track depths in Figure 5.

Material	Deep Rolling Pressure	Logarithmic Regressions	Coefficient of Determination R²
S235 FP	400 bar	T_d_ = 0.0127 ln(n) + 0.0265	0.9851
S235 FP	200 bar	T_d_ = 0.0115 ln(n) + 0.0260	0.9847
S235 FP	100 bar	T_d_ = 0.0104 ln(n) + 0.0277	0.9937
100Cr6 SA	400 bar	T_d_ = 0.0104 ln(n) + 0.0153	0.9842
100Cr6 SA	200 bar	T_d_ = 0.0089 ln(n) + 0.0173	0.9774
100Cr6 SA	100 bar	T_d_ = 0.0078 ln(n) + 0.0175	0.9929
C45 MPIE QT	400 bar	T_d_ = 0.0109 ln(n) + 0.0125	0.9569
C45 MPIE QT	200 bar	T_d_ = 0.0102 ln(n) + 0.0100	0.9421
C45 MPIE QT	100 bar	T_d_ = 0.0083 ln(n) + 0.0117	0.9587
100Cr6 MPIE QT1	400 bar	T_d_ = 0.0039 ln(n) + 0.0068	0.9747
100Cr6 MPIE QT1	200 bar	T_d_ = 0.0034 ln(n) + 0.0065	0.9957
100Cr6 MPIE QT1	100 bar	T_d_ = 0.0030 ln(n) + 0.0052	0.9386
100Cr6 QT1	400 bar	T_d_ = 0.0002 ln(n) + 0.0013	0.8397

**Table 7:** Material states from [15] and [16] used for comparison (calculation of HV values according to [23]).

		Hardening Parameter	Quenching Parameter	Tempering Parameter	Resulting Hardness	Approximate HV Values
**100Cr6**	H	850 °C (1 h)	-	-	66.0 ± 0.16 HRC	≈ 858 HV
	QT180	850 °C (1 h)	Oil 60 °C	180 °C (2 h)	62.4 ± 0.36 HRC	≈ 763 HV
	QT450	850 °C (1 h)	Oil 60 °C	450 °C (2 h)	49.4 ± 0.08 HRC	≈ 500 HV
**42CrMo4**	QT400	850 °C (2 h)	Oil 60 °C	400 °C (4 h)	47.0 ± 2.00 HRC	≈ 465 HV

## Abbreviations

*d*_b_	[mm]	tool diameter
E	[GPa]	Young’s modulus
f	[m/min]	feed rate
*F*_r_, *F*_r, theor._	[N]	deep rolling force, theoretical deep rolling force
h	[mm]	profile height
l	[mm]	profile length
n	[-]	number of rollovers
*p*_r_	[bar]	deep rolling pressure
R^2^	[-]	determination coefficient
Ra, Rz	[µm]	surface roughness
t_d_	[µm]	track depth
T_d_	[µm/(bar*mm)]	specific track depth
t_d_*	[µm]	elastic-plastic track depth during tool contact
t_w_	[mm]	track width
t_w_*	[mm]	elastic-plastic track width during tool contact
v_r_	[mm/min]	rolling speed v_r_
ν	[-]	poisson’s ratio
σ	[various]	standard deviation
σ_vgl_	[MPa]	max. equivalent stress σ_vgl_
